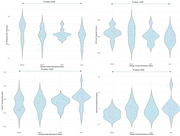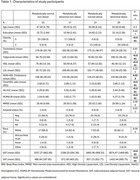# Brain Structural Changes and Amyloid Burden Linked to Midlife Obesity and Insulin Resistance: Implications for Alzheimer's Disease Risk

**DOI:** 10.1002/alz70855_098965

**Published:** 2025-12-23

**Authors:** Soheil Mohammadi, Mahsa Dolatshahi, Paul K. Commean, Farzaneh Rahmani, Caitlyn Nguyen, LaKisha Lloyd, Mahshid Naghashzadeh, Sara Hosseinzadeh Kasani, Bettina Mittendorfer, Claude Sirlin, Tammie L.S. Benzinger, Joseph E. Ippolito, John C. Morris, Cyrus A. Raji

**Affiliations:** ^1^ Mallinckrodt Institute of Radiology, Washington University in St. Louis, St. Louis, MO, USA; ^2^ Mallinckrodt Institute of Radiology, St. Louis, MO, USA; ^3^ Missouri University School of Medicine, Columbia, MO, USA; ^4^ University of California, San Diego, La Jolla, CA, USA; ^5^ Mallinckrodt Institute of Radiology, Washington University School of Medicine, St. Louis, MO, USA; ^6^ Washington University in St. Louis, St. Louis, MO, USA

## Abstract

**Background:**

Midlife obesity and insulin resistance are established risk factors for Alzheimer's disease, but their impact on brain structure and amyloid and tau burden remains unclear. This study explored the associations of obesity (BMI ≥ 30 kg/m²) and insulin resistance (HOMA‐IR) with cortical thickness, brain volume, and PET‐derived amyloid and tau burdens in Alzheimer's‐related regions.

**Method:**

Eighty cognitively normal, middle‐aged participants (mean age: 48.87 years; 32.5% male; 48.75% obese; mean BMI: 31.52 kg/m²) underwent 3T MRI, amyloid and tau PET scans, and metabolic assessments. Insulin resistance was defined as HOMA‐IR ≥ 1.9. Brain regions were segmented using FreeSurfer 7.1.1 with quality control. Amyloid PET imaging used ∼15 mCi [11C] PiB and computed whole‐brain amyloid Centiloid from the 30–60 minute scan window. Tau PET scans used AV‐1451 (7.2–10.8 mCi), analyzing standardized uptake value ratios (SUVRs) from the 50–70 minute window via the PET Unified Pipeline (PUP) Participants were categorized into four groups based on HOMA‐IR and obesity status: metabolically normal non‐obese (MNNO), metabolically abnormal non‐obese (MANO), metabolically normal obese (MNO), and metabolically abnormal obese (MAO). One‐way ANOVA, with age and sex as covariates, evaluated differences in Alzheimer's‐related cortical thickness and volume.

**Result:**

One‐way ANOVA identified significant between‐group differences in the right posterior cingulate volume, right superior temporal volume, and total temporal lobe volume. Post‐hoc ANCOVA revealed significantly lower right posterior cingulate volume in the MAO group compared to the MNNO group. Cortical thickness analyses showed significant differences in the left superior temporal and left temporal pole regions, with post‐hoc results indicating significantly lower left temporal pole thickness in the MAO compared to the MNNO group. PET‐derived results showed significant between‐group differences in whole‐brain amyloid Centiloid and total inferior temporal cortex amyloid. Post‐hoc ANCOVA revealed significantly higher whole‐brain amyloid Centiloid and total inferior temporal cortex amyloid in the MAO group compared to the MNNO group. No significant between‐group differences in tau burden were observed in the ROIs.

**Conclusion:**

Midlife obesity is associated with reduced volumes and higher amyloid burden in Alzheimer's disease‐associated region of the brain. The lack of significant tau burden differences reflects the cohort's subclinical stage of the disease.